# Prognostic impact of the post-treatment T cell composition and spatial organization in soft tissue sarcoma patients treated with neoadjuvant hyperthermic radio(chemo)therapy

**DOI:** 10.3389/fimmu.2023.1185197

**Published:** 2023-05-16

**Authors:** Luise Rupp, Antonia Resag, Vlatko Potkrajcic, Verena Warm, Rebekka Wehner, Korinna Jöhrens, Hans Bösmüller, Franziska Eckert, Marc Schmitz

**Affiliations:** ^1^ Institute of Immunology, Faculty of Medicine Carl Gustav Carus, Technische Universität (TU) Dresden, Dresden, Germany; ^2^ Department of Radiation Oncology, University Hospital Tuebingen, Tuebingen, Germany; ^3^ Institute of Pathology, University Hospital Tuebingen, Tuebingen, Germany; ^4^ National Center for Tumor Diseases (NCT), Partner Site Dresden, Dresden, Germany; ^5^ German Cancer Consortium (DKTK), Partner Site Dresden, and German Cancer Research Center (DKFZ), Heidelberg, Germany; ^6^ Institute of Pathology, University Hospital Carl Gustav Carus, Dresden, Germany; ^7^ Department of Radiation Oncology, Medical University of Vienna, Comprehensive Cancer Center Vienna, Vienna, Austria

**Keywords:** soft tissue sarcoma, immune microenvironment, T cells, immune modulation, radiotherapy, chemotherapy, hyperthermia

## Abstract

Soft tissue sarcomas (STS) form a heterogeneous group of tumors sharing a mesenchymal origin. Despite good local control of the disease, the occurrence of distant metastases often limits survival of STS patients with localized, high-risk tumors of the extremities. Accumulating evidence suggests a central role for the tumor immune microenvironment in determining the clinical outcome and response to therapy. Thus, it has been reported that STS patients with a high immune signature and especially presence of B cells and tertiary lymphoid structures display improved overall survival and response to checkpoint inhibitor treatment. Here, we explored the effect of curative multimodal therapy on the T cell landscape of STS using multiplex immunohistochemistry. We analyzed the phenotype, frequency, and spatial distribution of STS-infiltrating CD8^+^ T cells by staining for CD8, 4-1BB, Granzyme B, Ki67, PD-1, and LAG-3 as well as CD3^+^ T helper cells using a panel consisting of CD3, T-bet, GATA3, RORγT, FoxP3, and Ki67. All patients received neoadjuvant radiotherapy plus locoregional hyperthermia with or without chemotherapy. While the treatment-naïve biopsy sample allows an analysis of baseline T cell infiltration levels, both intra- and peritumoral areas of the matched resected tissue were analyzed to assess composition and spatial distribution of the T cell compartment and its therapeutic modulation. Generally, post-treatment tissues displayed lower frequencies of CD3^+^ and CD8^+^ T cells. Association with clinical data revealed that higher post-treatment frequencies of peritumoral and intratumoral CD3^+^ T cells and intratumoral PD-1^+^ CD8^+^ T cells were significantly associated with improved disease-free survival (DFS), while these densities had no prognostic significance in the biopsy. Upon spatial analysis, a high ratio of intratumoral to peritumoral CD8^+^ T cells emerged as an independent prognostic marker for longer DFS. These results indicate that the STS T cell landscape is altered by multimodal therapy and may influence the clinical outcome of patients. An enhanced understanding of the STS immune architecture and its modulation by neoadjuvant therapy may pave the way towards novel treatment modalities and improve the long-term clinical outcome of STS patients.

## Introduction

Soft tissue sarcomas (STS) comprise a heterogeneous group of rare tumors arising from extraskeletal mesenchymal tissue. They encompass a large variety of different molecular and histologic subtypes (1). Therapy is usually stratified according to three risk factors: tumor size, grading, and localization referring to the superficial fascia (2). Standard treatment of high-risk extremity tumors includes surgical resection and pre- or postoperative radiotherapy (RT) (3). While local control can be achieved in most cases, a significant proportion of patients develop distant metastases, especially in the lungs. Chemotherapy (CTX) is either applied concurrently or sequentially (4). Sequential chemotherapy seems to improve disease-free survival (DFS), but with limited efficacy, restricting its use to certain patient populations after interdisciplinary discussion (5).

Especially in the neoadjuvant setting, most extremity sarcomas are accessible for locoregional hyperthermia (RHT) which has been established as an additional treatment modality for high-risk STS (6). With electromagnetic applicators, the tumor area is heated to a temperature of 40-42°C for 60 min in conjunction with RT and/or CTX. High intratumoral temperatures have been described for sarcomas of the lower extremities (7). In a randomized phase 3 trial, RHT added to (neo)adjuvant CTX with etoposide, ifosfamide, and doxorubicin twice during each CTX cycle led to a significantly superior disease-free and disease-specific survival (8).

Although immunotherapies such as immune checkpoint inhibition have shown some effect in specific subtypes of STS, it has not become standard therapy and has not proven a breakthrough, neither in palliative treatment nor in the (neo)adjuvant setting. Certain subtypes seem to benefit more, such as scalp angiosarcoma, Kaposi’s sarcoma, alveolar soft part sarcoma, and undifferentiated pleomorphic sarcoma (9). Several studies demonstrated that the immune contexture of STS can predict the clinical outcome and response to immunotherapy (10, 11). Hence, a classification based on the gene expression profile revealed that STS with an immune-high signature, characterized by the presence of B cells and tertiary lymphoid structures, displayed improved survival and a high response rate to PD-1 checkpoint blockade (10). Evidence from various tumor entities, such as lung cancer or melanoma, further suggests that not only the frequency but also the phenotype, functional orientation, and spatial distribution of infiltrating T cells affect their clinical significance (12–14). Hence, T helper (Th) 1 cells are mainly associated with antitumor effects and improved clinical outcome, while Th2 cells and regulatory T cells (Tregs) are often linked to immunosuppressive properties and worse survival (15). Likewise, the functional state of tumor-infiltrating CD8^+^ T cells, which can be characterized by the expression of immune checkpoints and/or activation markers, may profoundly impact their clinical significance and predictive value. For example, a pan-cancer study by Zheng et al. demonstrated that patients with a high abundance of tissue-resident memory T cells and few exhausted T cells show better overall survival (OS) than tissue-resident memory T cell-low and exhausted T cell-high patients (16).

Generally, several factors such as molecular and histological subtype, anatomical site of the tumor, and especially previous therapy influence its immune contexture and clinical behavior. Especially in tumor types not responding to immunotherapy, strategies to convert poorly infiltrated tumors into immune-high tumors are crucial to improve the efficacy of immunotherapy and make it accessible for a wider range of patients. Classical cytotoxic, DNA-damaging therapy approaches like CTX and RT can induce or improve tumor-specific T cell responses and are thus being discussed to be used in combinational settings with immunotherapy (17–19). So far, studies focusing on the modulation of the tumor microenvironment (TME) by standard of care treatment regimens, including RHT, in matched STS samples are scarce (20). Here, we analyzed the frequency, composition, and spatial distribution of STS-infiltrating T cells prior to and after multimodal treatment. We focused on a group of patients sharing the occurrence of localized high-grade STS in the extremities as well as a similar treatment regimen consisting of RT and RHT with or without CTX. An in-depth analysis of the CD8^+^ and Th cell composition by multiplex immunohistochemistry (mIHC) in matched samples of biopsy and resected STS tissues allows assessing its treatment-mediated modulation and may pave the way towards the development of novel immunotherapeutic strategies.

## Material and methods

### Patient cohort and sample collection

STS patients (n = 115) treated at the Center for Bone and Soft Tissue Sarcoma at the University Hospital Tuebingen were screened for extremity STS, administered neoadjuvant multimodal therapy, and availability of pre- and post-treatment formalin-fixed paraffin-embedded (FFPE) tissue sections, resulting in 21 patients eligible for the cohort (**Supplementary Figure 1**). Myxoid liposarcomas were excluded due to their specific clinical behavior (radio- and chemosensitivity, pattern of metastases) and differing treatment concepts.

All patients had undergone initial incisional biopsy of the tumor and staging with at least local magnetic resonance imaging (MRI) and chest computed tomography to exclude pulmonary metastases. Multimodal therapy included surgery, neoadjuvant RT, and concomitant RHT for all patients of the cohort. Concomitant and/or sequential CTX was applied in selected patients (e.g. young patients with high-grade STS). RT was delivered using 3D conformal RT (n = 19) or intensity-modulated RT (n = 2) with standard dose of 50-50.4 Gy in 25-28 fractions. Concomitant CTX with ifosfamide was given in two cycles (3000 mg/m² on day 1 and day 2, as well as on day 21 and day 22 of the RT, n = 14). Sequential CTX was applied in selected patients (n = 12) in 3-4 cycles (ifosfamide 3000 mg/m^2^ and doxorubicin 60 mg/m^2^ every 22 days). RHT was delivered concomitantly to RT twice a week with or without magnetic resonance guidance (BSD 2000/3 D MRI, Pyrexar Medical, formerly BSD medical corporation, Salt Lake City, UT). Resection was conducted 9-70 days (median 41 days) after end of RT. The study was approved by the Ethics Committee of the Medical Faculty of the University of Tuebingen (901/2019BO2). Oncologic outcome data as well as clinical patient and tumor features were retrieved from the clinical records. Clinicopathologic information is summarized in **Table 1**.

**Table 1:** Patient characteristics of the STS cohort.

Age (years)		
Median	59	
Range	21-82	
Gender (n, %)		
Male	13	62%
Female	8	38%
Location (n, %)		
Upper extremity	5	24%
Lower extremity	16	76%
Tumour size (n, %)		
<5 cm	2	10%
5-10 cm	7	33%
>10 cm	12	57%
Grading according to FNCLCC (n, %)		
G2	10	48%
G3	11	52%
Histology (n, %)		
Liposarcoma	4	19%
Synovial sarcoma	2	10%
Leiomyosarcoma	3	14%
NOS	8	38%
Other^1^	4	19%
Concurrent Ifosfamide (n, %)		
Yes	14	67%
No	7	33%
Sequential Adriamycin/Ifosfamide (n, %)		
Yes	12	57%
No	9	43%
Resection status (n, %)		
R0	18	86%
R1	3	14%
Pathological response (n, %)^2^		
Yes	11	52%
No	10	48%

^1^One spindle cell sarcoma, one myxoid sarcoma without translocation, one myxofibrosarcoma, one malignant peripheral nerve sheath tumor.

^2^Defined as <10 % vital tumor.

FFPE material of 21 patients was retrieved from the biobank of the Tuebingen Institute for Pathology. However, one biopsy specimen was excluded afterwards due to insufficient quality of the sample resulting in 20 pre-treatment biopsy specimens and 21 post-treatment resection specimens for the final analysis. As biopsies were performed intralesionally, only intratumoral tissue was assessed. In the resection samples, marginal FFPE blocks were chosen to enable spatial analysis of T cell distribution, resulting in 19 peritumoral and 16 intratumoral tissues. Paired tissue samples before and after therapy were available for 16 patients (both intratumoral tissue) and resection samples harboring both peritumoral and intratumoral tissue were obtained from 14 patients. Areas subjected to analysis were annotated in HE sections by experienced pathologists. Necrotic areas and normal tissue were excluded in all samples.

### Multiplex immunohistochemistry

For the detection and quantification of tumor-infiltrating T cell subpopulations in STS, mIHC of FFPE tissue sections was performed on a Ventana Discovery Ultra instrument (Ventana Medical Systems, Basel, Switzerland) as described in detail previously (21). Staining was conducted with two different antibody panels each including six different primary antibodies utilizing the tyramid signal amplification-based OPAL technology (Akoya Biosciences, Marlborough, MA, USA). Starting the staining procedure, 2.5 µm thick FFPE tissue sections were deparaffinized and rehydrated at 69°C for 3x 8 min in EZ Prep solution (Ventana Medical Systems), followed by a heat-mediated antigen retrieval at 95°C for 32 min in Cell Conditioning Solution (CC) 1 (Ventana Medical Systems, pH 9). Subsequently, the primary antibody was manually applied to the tissue section and incubated at 36°C at an individual dilution and incubation period (**Supplementary Table 1**). The secondary antibody (Ventana Medical Systems) and optionally tertiary antibody (Ventana Medical Systems) was automatically applied and incubated for 12 min at 36°C followed by the incubation of the OPAL reagent (Akoya Biosciences) for 8 min at room temperature. Subsequent antibody denaturation was performed for 24 min at 100°C in CC2 (Ventana Medical Systems, pH 6). The antibody incubation procedure was repeated six times and finalized by counterstaining with DAPI for 4 min. Stained tissue sections were mounted in Fluoromount-G^®^ medium (SouthernBiotech, Birmingham, Alabama, USA) and stored at 4°C.

### Image analysis and cell quantification

Imaging of the stained FFPE slides was performed on the Vectra 3.0 Automated Imaging System (Akoya Biosciences). Whole slide scans at 100x magnification were obtained to define Regions of Interest (ROIs) using the Phenochart™ software (Akoya Biosciences). Based on the ROIs, multispectral images (MSIs) at 200x magnification were taken and subsequently analyzed using inForm software (Akoya Biosciences). Spectral unmixing of OPAL fluorophore signals was based on a manually built OPAL fluorophore library. Cell quantification was done in a semi-automated manner using manually trained algorithms to discriminate between tissue/non-tissue area, segment cells based on DAPI signal and finally, phenotype cells based on the staining intensity and pattern of the respective marker.

MSIs were exported as multi-channel TIFFs and processed in imageJ software for representative images (22). Upon importing the images as TIFF-virtual stacks, single channels were processed using arithmetic point operations. For figures showing a larger field of view, multiple images were stitched using the grid/collection stitching plugin (23).

### Statistical analysis

Data was analyzed using RStudio and R v4.2.1 (24). Prior to analysis, a Shapiro Wilk test was used to test for non-parametric data. Significances were determined using the Wilcoxon rank-sum test. Upset plots were generated with the R package ComplexUpset (25, 26). Beforehand, random downsampling was used to get unbiased phenotype distributions utilizing the groupdata2 package (27). One patient with an exceptionally low cell number was excluded from generating the upset plots. Univariate and multivariate survival analysis using the Kaplan-Meier method and the Cox proportional hazards (PH) model, respectively, was conducted with the packages survival (28, 29) and survminer (30). Visualization of the Cox PH model results as a forest plot was done with the forplo package (31). Scaled Schoenfeld residuals were used to confirm the proportional hazards assumption. Hierarchical cluster analysis of T cell densities was performed using the packages stats (24) to generate the patient dendrogram and ComplexHeatmap (32, 33) for heatmap visualization. Therefore, empirical percentile transformation was applied to receive comparable data and patients with similar T cell densities were determined by the complete linkage clustering method.

## Results

### Characterization of tumor-infiltrating CD8^+^ T cells and analysis of the T helper cell composition prior to and post multimodal therapy in soft tissue sarcomas

**Figure 1:**
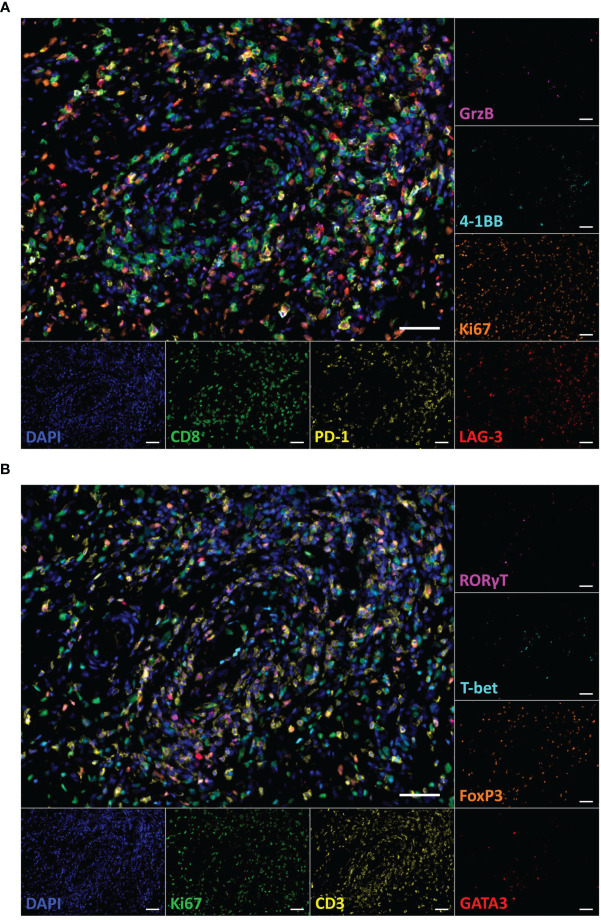
Multiplex immunohistochemistry staining of T cell subpopulations in a paraffin-embedded specimen of a dedifferentiated liposarcoma. **(A)** Representative multicolor image of the CD8^+^ T cell panel staining including the markers DAPI (blue), CD8 (green), 4-1BB (cyan), Granzyme (Grz) B (magenta), Ki67 (orange), LAG-3 (red), and PD-1 (yellow). **(B)** Representative image of the Th panel staining including the markers DAPI (blue), CD3 (yellow), T-bet (cyan), GATA3 (red), RORγT (magenta), FoxP3 (orange), and Ki67 (green). Both images show corresponding tissue sections. Scale bars indicate 50 µm.

**Figure 2:**
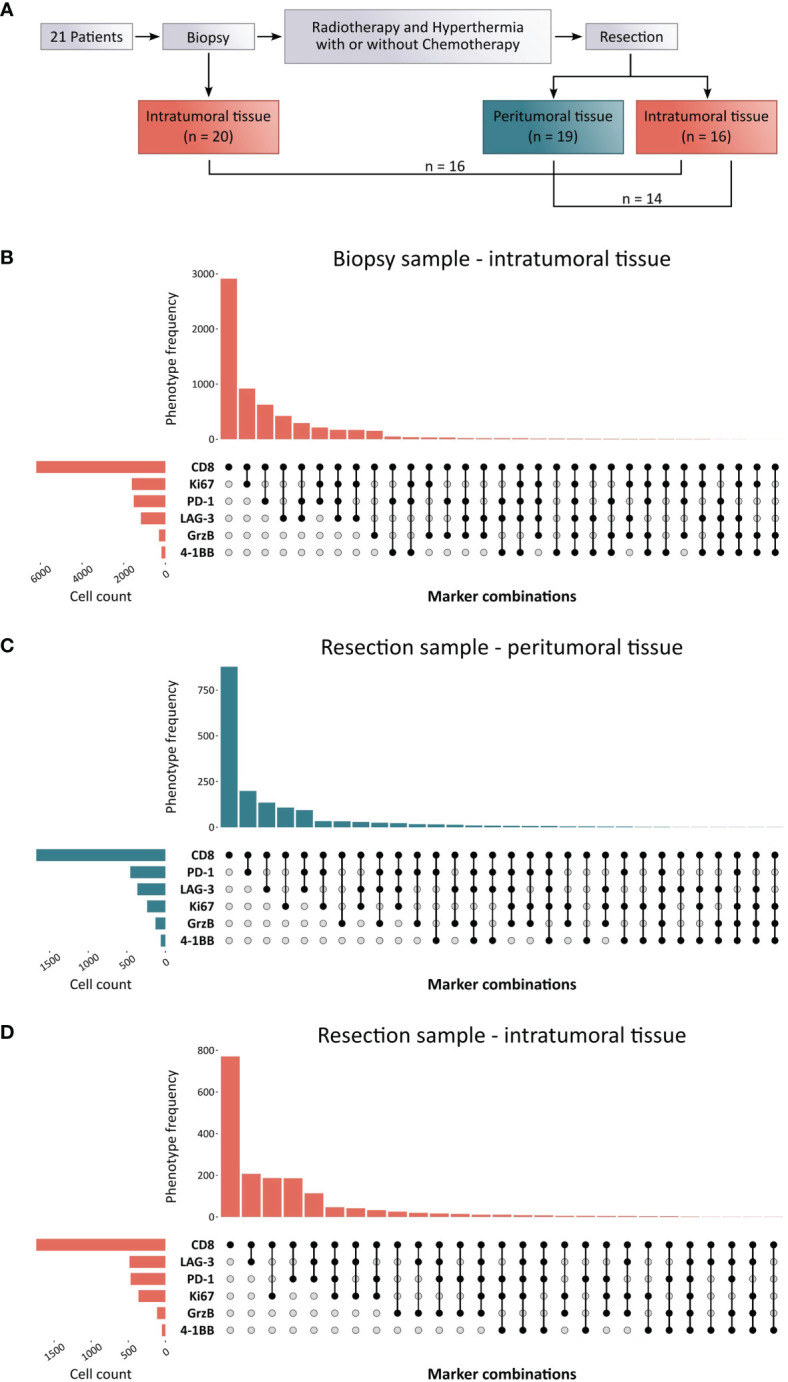
Experimental layout and CD8^+^ phenotype distribution in biopsy and resected soft-tissue sarcoma (STS) samples. **(A)** Schematic overview of the sample collection process. The cohort included 21 STS patients of which pre-treatment samples were available from the biopsy as well as post-treatment samples from the resection specimen. All patients received a multimodal therapy regimen consisting of radiotherapy and regional hyperthermia with or without concomitant and sequential chemotherapy. **(B–D)** Upset plots from down sampled raw data representing the most common marker combinations resulting from the CD8^+^ T cell panel as well as their frequency (upper bar chart) within **(B)** the intratumoral tissue of the biopsy sample, **(C)** the peritumoral tissue, and **(D)** the intratumoral tissue of the resection sample. The left bar chart visualizes the cell number of marker-positive T cells.

To assess the localization, frequency, and functional orientation of T cells in high-grade STS patients, mIHC staining of two 7-color panels was employed. For characterization of CD8^+^ T cells, the markers CD8, 4-1BB, Granzyme (Grz) B, Ki67, PD-1, and LAG-3 were evaluated in addition to DAPI nuclear staining (**Figure 1A**). Furthermore, the differentiation of Th cells was explored by staining for CD3, Ki67, and the master transcription factors T-bet, GATA3, RORγT, and FoxP3 (**Figure 1B**). In order to gain a comprehensive view of the T cell landscape in STS and its alteration by multimodal therapy, we analyzed three types of samples in a total of 21 patients (**Figure 2A**). A biopsy that was taken prior to any treatment allows the evaluation of the naïve tumor immune microenvironment while both intratumoral and peritumoral areas were evaluated in the post-treatment sample. Importantly, simultaneous imaging of six markers enables co-detection of both activating and inhibiting molecules on the same cell. The CD8^+^ phenotype frequency and the distribution of coexpressed markers in the biopsy sample as well as peritumoral and intratumoral areas of the resected tissue are illustrated in **Figures 2B–D**. Strikingly, CD8^+^ T cells negative for all functional markers represent the major population in all three sample types. While Ki67^+^ CD8^+^ T cells take the second largest share in the biopsy, CD8^+^ T cells expressing the immune checkpoint molecules LAG-3 or PD-1 are more frequent in the resected tissue (**Figures 2C, D**). Consistently, CD8^+^ T cells expressing GrzB, which is involved in the direct killing of target cells, or the costimulatory molecule 4-1BB represent the least frequent populations before and after therapy in both peritumoral and intratumoral tissue.

**Figure 3:**
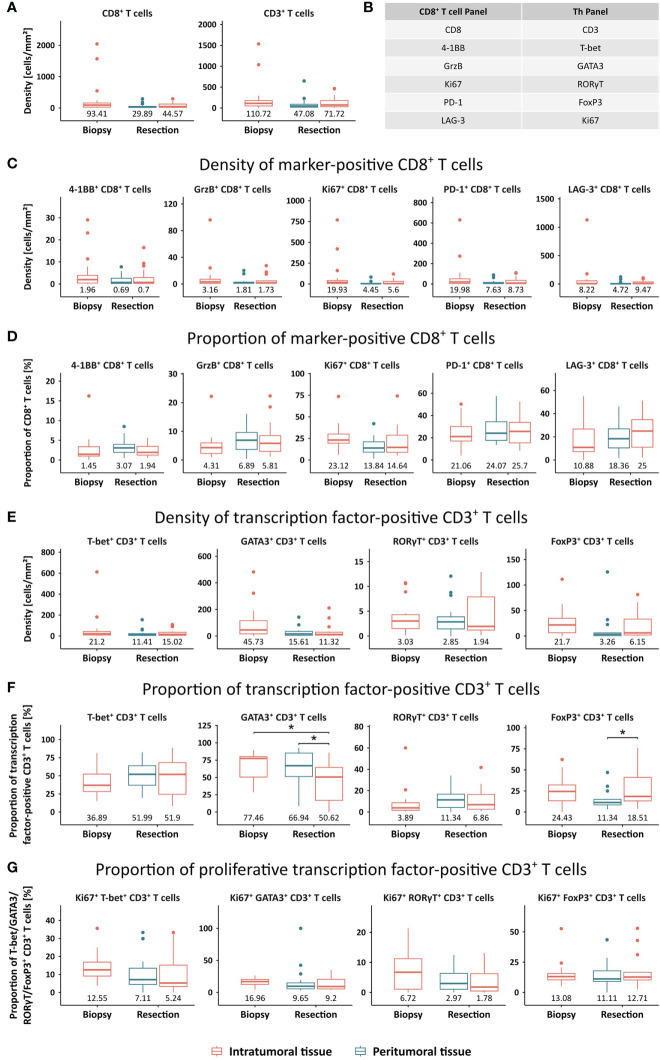
Quantification of T cell phenotypes in STS, compared between the intratumoral tissue of the biopsy sample and the peritumoral and intratumoral area of the resection sample. **(A)** Densities [cells/mm²] were assessed for CD8^+^ and CD3^+^ T cells. **(B)** Overview of all markers included in the CD8^+^ T cell panel and the Th panel that resulted in the definition of several T cell phenotypes. **(C, D)** Densities [cells/mm²] and proportions of CD8^+^ T cells positive for a functional marker. **(E, F)** Densities [cells/mm²] and proportions of CD3^+^ T cells positive for a transcription factor. **(G)** Proportion of proliferative transcription factor-positive CD3^+^ T cells, determined by the expression of Ki67. Median values are displayed. Significant differences were determined using the unpaired Wilcoxon test and are shown as * ≙ p-value ≤ 0.05.

In further studies, cell densities of various T cell populations were compared between pre- and post-therapy samples (intratumoral) as well as post-treatment peritumoral and intratumoral tissue (**Figure 3**). Interestingly, a reduction of the median CD8^+^ T cell density was observed from 93.41 cells/mm² in the biopsy to 29.89 cells/mm² and 44.57 cells/mm² in the peritumoral and intratumoral area of the resected tissue, respectively (**Figure 3A**). Similarly, a reduced number of CD3^+^ T cells was detected in post-treatment tissues in comparison to pre-treatment biopsies. When only considering matched samples, no significant difference between CD3^+^ or CD8^+^ T cell numbers in pre- and post-therapy samples as well as peritumoral and intratumoral tissue was observed (**Supplementary Figure 2**). Based on the markers included in the two panels that were stained (**Figure 3B**), main phenotypes were analyzed in terms of cell density and proportion of the parent population (**Figures 3C–G**). For example, the population of 4-1BB^+^ CD8^+^ cells is comprised of all cells that are positive for CD8 and 4-1BB, irrespective of coexpression of the other four markers. With a median of 1.96 cells/mm² and 3.16 cells/mm² respectively, the densities of 4-1BB^+^ CD8^+^ and GrzB^+^ CD8^+^ T cells were low in the biopsy and further reduced in the resected sample with no difference between peritumoral and intratumoral area (**Figure 3C**). Proliferative Ki67^+^ CD8^+^ T cells displayed a higher density of 19.93 cells/mm² in the biopsy sample, while lower numbers were detected in the post-treatment specimen (4.45 cells/mm² in the peritumoral and 5.6 cells/mm² in the intratumoral area). CD8^+^ T cells expressing PD-1 or LAG-3 showed an abundance of 19.98 cells/mm² and 8.22 cells/mm², respectively, in the biopsy tissue. Similarly, the frequencies of these populations in the resected tissue were lower, with slightly increased numbers in the intratumoral tissue (8.73 cells/mm² PD-1^+^ CD8^+^ and 9.47 cells/mm² LAG-3^+^ CD8^+^) in comparison to the peritumoral area (7.63 cells/mm² PD-1^+^ CD8^+^ and 4.72 cells/mm² LAG-3^+^ CD8^+^). Subsequently, the relative proportions of these populations of the total CD8^+^ T cell numbers were compared (**Figure 3D**). In concordance with the lowest cell densities, the populations of 4-1BB^+^ CD8^+^ and GrzB^+^ CD8^+^ cells hold the lowest share of the CD8^+^ T cell parent population. In contrast, CD8^+^ T cells coexpressing Ki67, PD-1, or LAG-3 comprise 23.12%, 21.06%, and 10.88% of all CD8^+^ T cells in the biopsy, respectively. Overall, the proportion of Ki67^+^ CD8^+^ cells slightly decreased upon multimodal therapy, while all other populations displayed a slight increase.

In line with CD8^+^ T cells, all analyzed CD3^+^ Th cell subsets displayed lower median frequencies in the resection specimens in comparison to the biopsy (**Figure 3E**). To determine relative proportions of different Th cell subsets, CD3^+^ cells that are positive for at least one of the four transcription factors were used as the parent population (**Figure 3F**). GATA3^+^ CD3^+^ cells account for the largest proportion in the biopsy sample, followed by T-bet^+^ CD3^+^, FoxP3^+^ CD3^+^, and RORγT^+^ CD3^+^ cells with median proportions of 77.46%, 36.89%, 24.43%, and 3.89%, respectively. Upon therapy, a slight redistribution of Th subsets was observed with a significantly reduced share of GATA3^+^ CD3^+^ T cells and increased proportions of RORγT^+^ CD3^+^ as well as T-bet^+^ CD3^+^ T cells. While GATA3^+^ CD3^+^ T cells had a significantly higher proportion in the peritumoral compared to the intratumoral area, the proportion of FoxP3^+^ CD3^+^ T cells showed a significant inverse distribution. Finally, proliferative proportions of each Th cell subset were defined by coexpression of Ki67 (**Figure 3G**). In the biopsy, GATA3^+^ CD3^+^ T cells displayed the highest median proportion of proliferative cells, followed closely by FoxP3^+^ CD3^+^ and T-bet^+^ CD3^+^ T cells. While this relative amount of Ki67^+^ cells stayed roughly the same in the resected post-therapy specimen for FoxP3^+^ CD3^+^ T cells, it was moderately reduced in all other analyzed Th subsets.

### Increased frequencies of GrzB^+^ CD8^+^ and RORγT^+^ CD3^+^ T cells are associated with higher tumor grading

**Figure 4:**
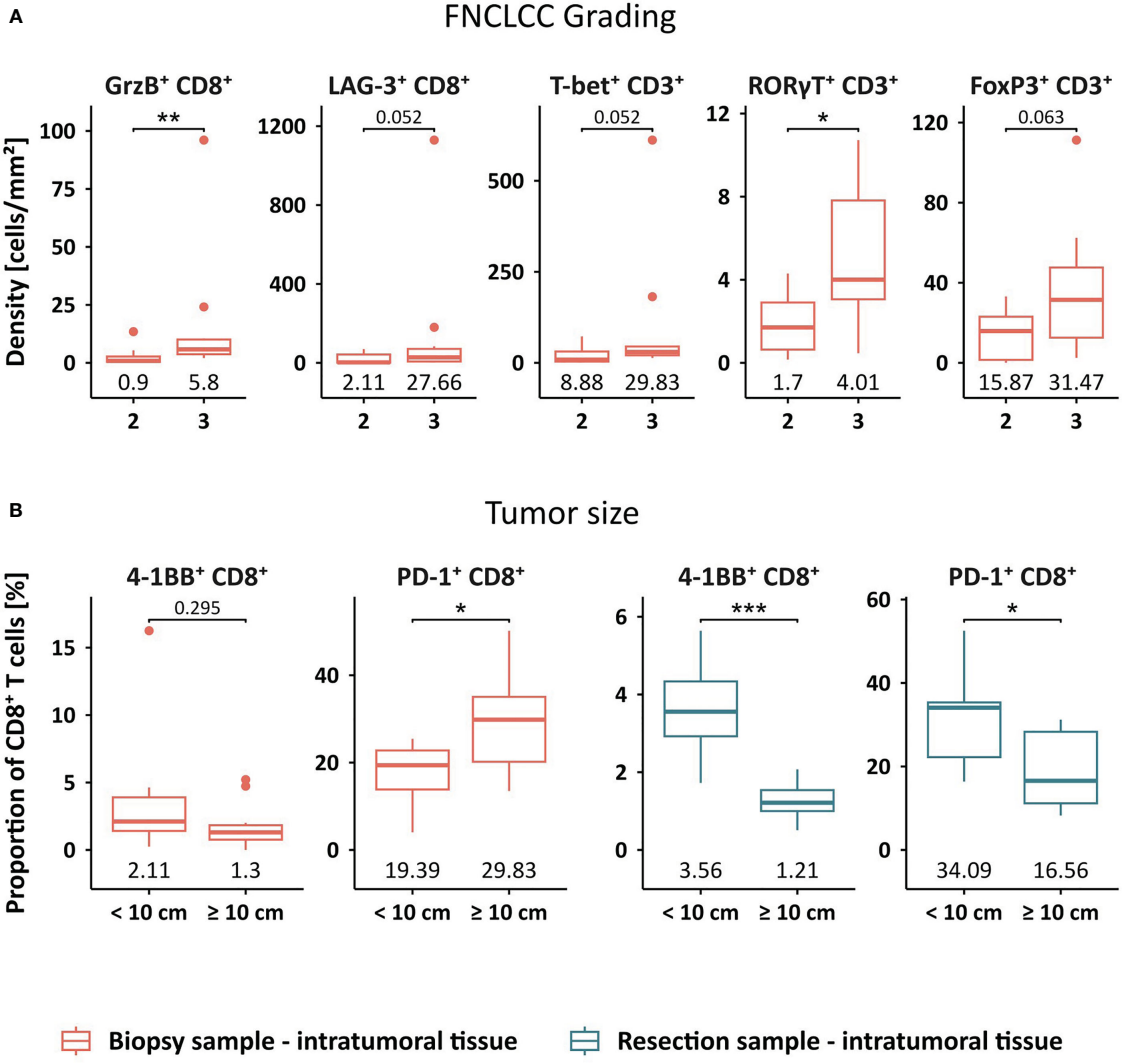
Clinical association of STS-infiltrating T cell phenotypes. **(A)** Cell densities [cells/mm²] of intratumoral GrzB^+^ CD8^+^, LAG-3^+^ CD8^+^, T-bet^+^ CD3^+^, RORγT^+^ CD3^+^, and FoxP3^+^ CD3^+^ T cells in the pre-treatment sample compared between grade 2 and grade 3 STS. **(B)** Proportion of 4-1BB^+^ CD8^+^ T cells and PD-1^+^ CD8^+^ T cells in the pre- and post-treatment sample compared between smaller and larger STS, stratified by the size of 10 cm maximum diameter. Significant differences were determined using the unpaired Wilcoxon test and are shown as * ≙ p-value ≤ 0.05, ** ≙ p-value ≤ 0.01, and *** ≙ p-value ≤ 0.001.

Next, we evaluated a potential link between the abundance of tumor-infiltrating T cells and clinicopathological characteristics, such as grading and tumor size (**Figure 4**). We found that the density of GrzB^+^ CD8^+^ and RORγT^+^ CD3^+^ T cells was significantly higher in grade 3 STS compared to grade 2 tumors (**Figure 4A**). Furthermore, a trend towards higher frequencies of LAG-3^+^ CD8^+^, T-bet^+^ CD3^+^, and FoxP3^+^ CD3^+^ T cells in higher grade STS tissues was observed. Moreover, smaller tumors with a maximum diameter below 10 cm displayed higher proportions of 4-1BB^+^ and PD-1^+^ CD8^+^ T cells in the resection sample compared to tumors larger than 10 cm (**Figure 4B**). In contrast, a correlation of a higher PD-1^+^ CD8^+^ proportion to larger tumor size occurred in the biopsy sample while the proportion of 4-1BB^+^ CD8^+^ T cells did not differ between both groups. Further results comparing frequencies of all main populations of the CD8^+^ and CD3^+^ T cell subsets are listed in **Supplementary Tables 2, 3**.

### The post-treatment T cell landscape defines clinical outcome of STS patients

**Figure 5:**
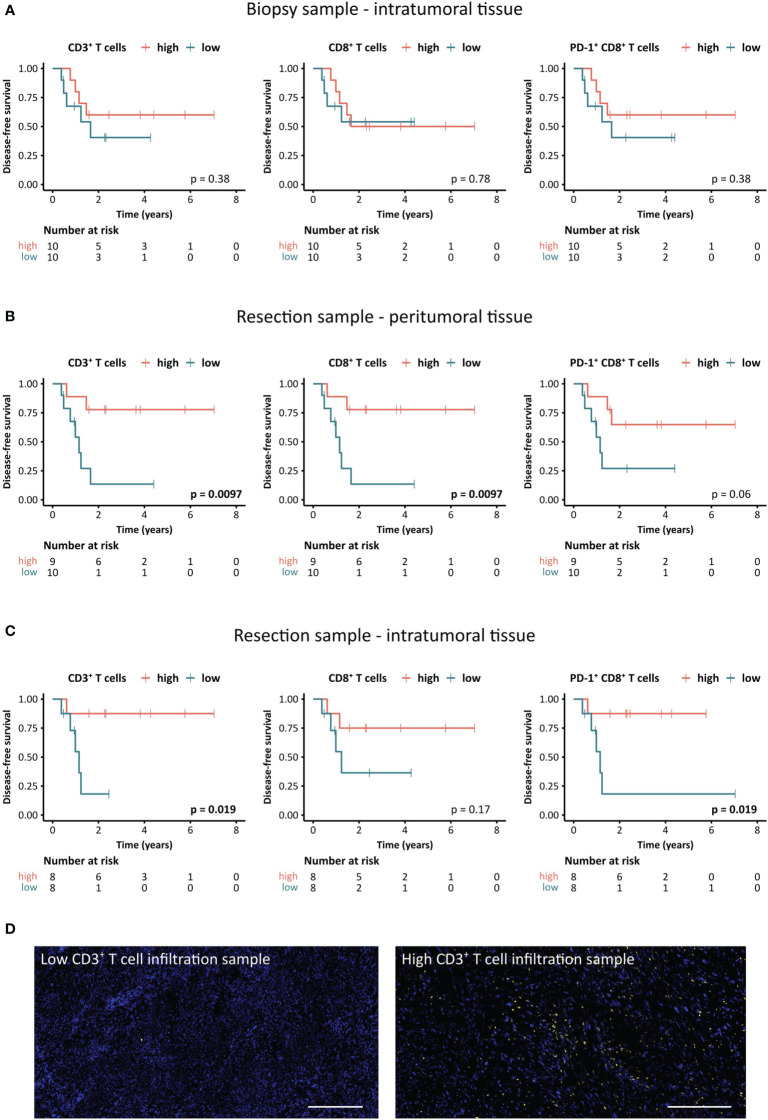
Association between STS-infiltrating T cells and disease-free survival (DFS) of STS patients. **(A–C)** Kaplan-Meier survival analysis of DFS stratified by CD3^+^, CD8^+^, and PD-1^+^ CD8^+^ T cell densities **(A)** in the intratumoral tissue of the biopsy sample as well as **(B)** the peritumoral and **(C)** the intratumoral tissue of the resection sample. Patients were stratified based on the median cell density [cells/mm²]. Log-rank test was performed and p-values ≤ 0.05 were considered significant. **(D)** Representative images of the CD3^+^ T cell staining in specimens with the lowest and highest density of CD3^+^ T cells (yellow), respectively. Scale bars indicate 300 µm.

We further investigated whether the frequency of tumor-infiltrating T cells is associated with the DFS of STS patients (**Figure 5**). While the density of CD3^+^, CD8^+^, or PD-1^+^ CD8^+^ T cells in the biopsy did not correlate with DFS (**Figure 5A**), these frequencies showed prognostic value in peritumoral and intratumoral areas of the resected tissue (**Figures 5B, C**). In the peritumoral area, higher frequencies of CD3^+^ or CD8^+^ T cells were linked to significantly longer DFS (p=0.0097), while an increased density of PD-1^+^ CD8^+^ T cells showed a strong trend towards a positive impact on DFS (p=0.06) (**Figure 5B**). Moreover, higher numbers of CD3^+^ T cells or PD-1^+^ CD8^+^ T cells within the intratumoral area of the resected sample significantly correlated with improved DFS (p=0.019) (**Figure 5C**). Representative images of the patient with the lowest frequency of CD3^+^ T cells and the patient with the highest intratumoral CD3^+^ T cell density in the resection specimen are shown in **Figure 5D**.

**Figure 6:**
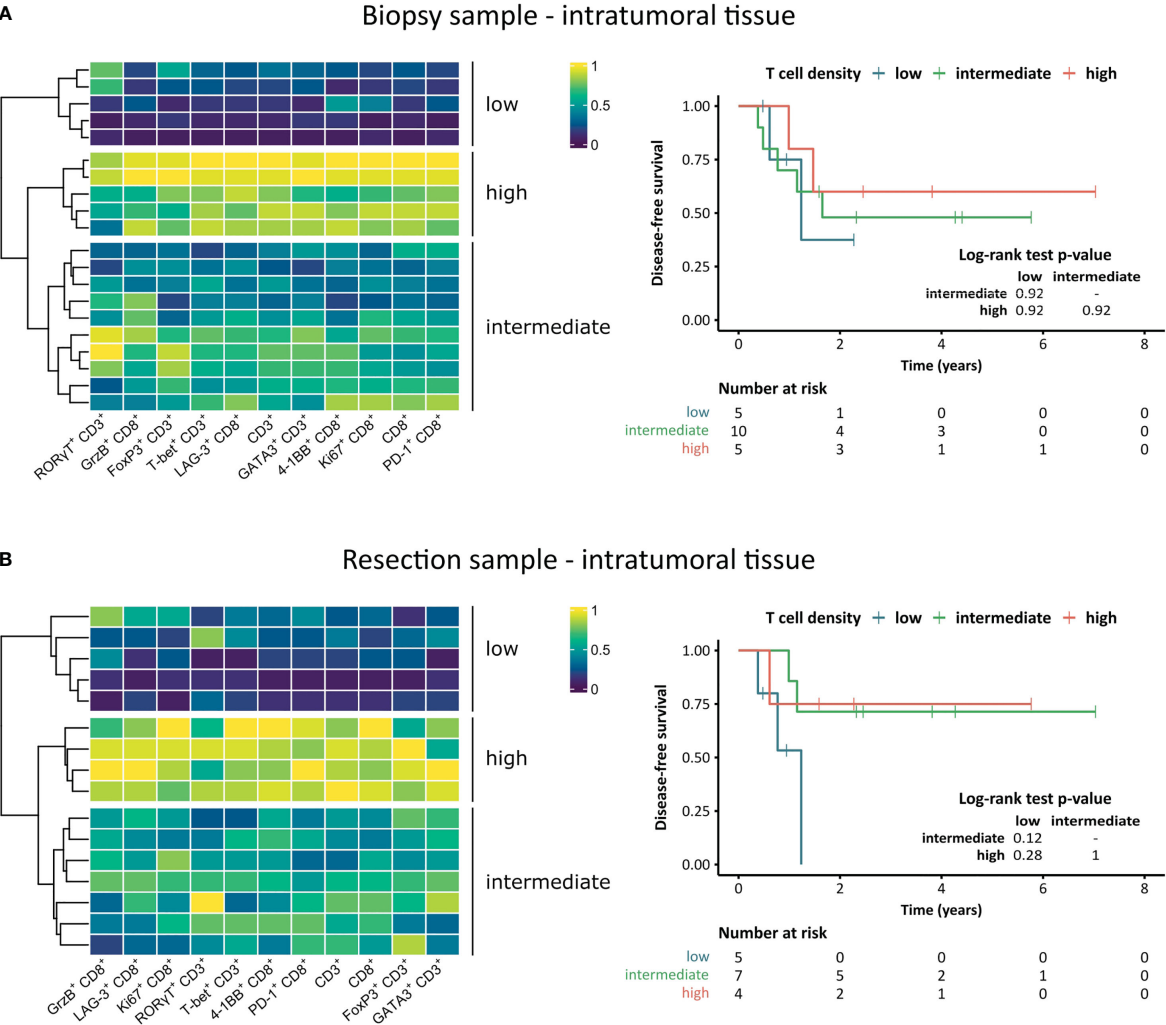
Unsupervised hierarchical clustering heatmaps and association of clusters to disease-free survival of STS patients. **(A, B)** Heatmaps based on intratumoral T cell densities including hierarchical clustering dendrograms and Kaplan-Meier survival analysis of the patient clusters for **(A)** pre-treatment and **(B)** post-treatment STS. Pairwise log-rank tests were performed using the Benjamini-Hochberg method for p-value adjustment, p-values ≤ 0.05 were considered significant.

To classify the patient cohort according to T cell infiltration levels, we performed an unsupervised hierarchical clustering based on the densities of the main populations of both the CD8^+^ and CD3^+^ T cell panel (**Figure 6**). In the intratumoral area of both the biopsy and the resected tissue, clusters of T cell-low, -intermediate, and -high groups formed. To assess a potential link to distinct clinical behavior of these groups, differences in DFS were determined using Kaplan-Meier graphs and log-rank tests. While there was no difference between the three groups in the biopsy (**Figure 6A**), the T cell-low cluster displayed shorter DFS compared to patients in the intermediate and high cluster in the intratumoral compartment of the resection specimen (**Figure 6B**).

**Figure 7:**
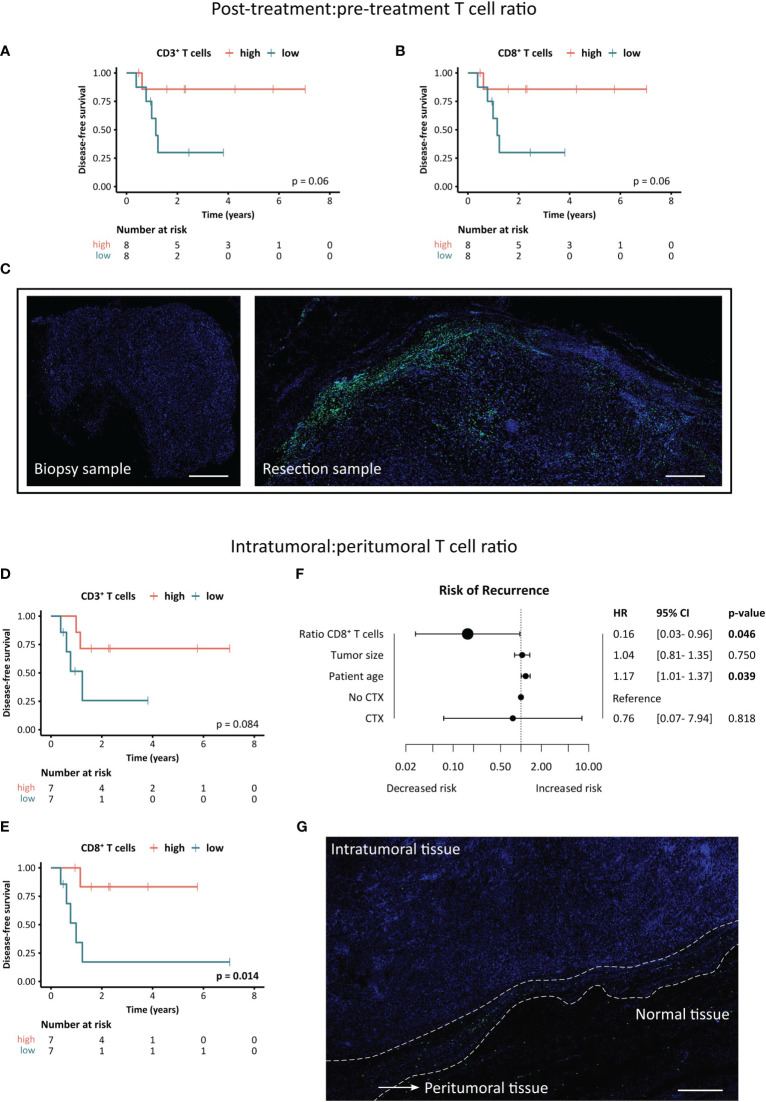
Clinical significance of post- to pre-treatment and intratumoral to peritumoral T cell ratios. **(A–C)** Association between post-treatment:pre-treatment T cell ratio and disease-free survival of STS patients. **(A, B)** Kaplan-Meier survival analysis of **(A)** CD3^+^ T cell ratios and **(B)** CD8^+^ T cell ratios. Patients were stratified based on the median ratio between post-treatment and pre-treatment intratumoral T cell density with higher ratios indicating a higher post-treatment T cell infiltration. Log-rank test was performed and p-values ≤ 0.05 were considered significant. **(C)** Representative images of the CD8^+^ T cell staining in the biopsy (pre-treatment) and resection sample (post-treatment) of the patient exhibiting the highest T cell ratio. Scale bars indicate 500 µm. **(D–G)** Association between intratumoral:peritumoral T cell ratio and disease-free survival of STS patients. **(D, E)** Kaplan-Meier survival analysis of **(D)** CD3^+^ T cell ratios and **(E)** CD8^+^ T cell ratios. Patients were stratified based on the median ratio between intratumoral and peritumoral T cell density (post-treatment) with higher ratios indicating a higher intratumoral T cell infiltration. Log-rank test was performed and p-values ≤ 0.05 were considered significant. **(F)** Forest plot of multivariate Cox proportional hazards (PH) regression model for the risk of recurrence. Hazard ratios (HR) and 95% confidence intervals (CI) are shown. Log-rank test was performed and p-values ≤ 0.05 were considered significant. **(G)** Representative image of a resection specimen exhibiting the lowest intratumoral:peritumoral T cell ratio. Scale bar indicates 500 µm.

To assess the effect of multimodal therapy on the T cell landscape, we determined the post- to pre-treatment ratio of intratumoral T cells in paired samples (**Figures 7A–C**). Interestingly, high ratios of both CD3^+^ and CD8^+^ T cells showed a strong trend towards a positive impact on DFS (p=0.06) (**Figures 7A, B**). A representative image of matched pre- and post-therapy samples displaying higher CD3^+^ T cell infiltration in the resected tissue is shown in **Figure 7C**.

Next, we investigated the spatial distribution of T cells in the STS TME by evaluating the ratio of densities in the intratumoral to peritumoral area of the resected post-treatment tissue (**Figures 7D–G**). A higher ratio of intratumoral to peritumoral CD3^+^ T cells showed a trend towards a positive impact on DFS (p=0.084), while an increased ratio of CD8^+^ T cells was associated with significantly better clinical outcome (p=0.014) (**Figures 7D, E**). To adjust for other clinicopathological covariates, a multivariate cox PH analysis was performed (**Figure 7F**). The multivariate analysis revealed that a higher ratio of intratumoral to peritumoral CD8^+^ T cells was significantly and independently correlated to a lower risk of recurrence (HR=0.16, p=0.046). **Figure 7G** shows a representative image of the patient with the lowest ratio of intratumoral to peritumoral CD8^+^ T cell infiltration, demonstrating an excluded TME type.

## Discussion

The frequency and spatial distribution of tumor-infiltrating T cells play a crucial role for the antitumor immune response and clinical outcome of cancer patients. Additionally, an improved understanding of the T cell landscape enables the development and refinement of effective treatment strategies (34). Due to the scarcity and heterogeneity of STS, studies investigating STS-infiltrating T cells are limited and mainly based on heterogeneous cohorts, comprising different histological and molecular subtypes as well as treatment regimens. In this study, we dissected the T cell infiltration landscape of localized, high-risk extremity STS undergoing curative, multimodal treatment using two 7-color mIHC panels to quantify tumor-infiltrating T cells and assess the functional status of CD8^+^ T cells as well as the polarization of Th cells. A carefully selected STS patient cohort allowed for the comparison of T cell infiltration before and after multimodal neoadjuvant therapy consisting of RT and RHT with or without CTX. Moreover, the concomitant analysis of post-treatment intratumoral and peritumoral tissue enabled novel insights into the spatial distribution of CD8^+^ and Th cells in STS and their effect on DFS.

Conventional anticancer treatments, including RT and CTX, harbor immunomodulatory properties and have the potential to prime the immune TME in addition to immunotherapy, which is subject to a growing number of clinical studies (35, 36). Also RHT, a frequently used treatment for extremity STS, was reported to show immunogenic effects (37). Boosting the pre-existing immune TME is of particular interest for STS, as they generally exhibit a low to moderate T cell infiltration compared to other tumor entities with major differences between STS subtypes (38–42). However, in our study, CD3^+^ and CD8^+^ T cell densities showed a trend to decrease following neoadjuvant therapy. Utilizing IHC and quantifying T cells *via* scoring by a pathologist, others reported that conventional therapy did not affect CD8^+^ T cell infiltration levels. Snow et al. observed this in a cohort of liposarcoma patients after RT (43), and Issels et al. in a mixed STS cohort after CTX with or without RHT (20). Importantly, the study by Snow et al. contained mixed tumor localizations including retroperitoneal and extremity liposarcomas, as well as grades 1-3, of which low-grade tumors accounted for more than 50% of patients. Moreover, other studies indicate that RT may lead to an increased T cell infiltration in STS (44, 45). In the study by Keung et al., 17 matched undifferentiated pleomorphic sarcoma tissues were analyzed, consisting of mixed trunk and extremity tumors. In another STS cohort of 19 matched tissues, a strong trend towards an increased CD3^+^ T cell infiltration after neoadjuvant treatment with RT and/or CTX using mIHC was reported (46). However, transcriptomic analysis of a cohort including these patients showed no significant difference between pre- and post-treatment CD3^+^ and CD8^+^ T cell frequencies. This study also contains tumors of mixed extremity, central, and retroperitoneal location as well as STS grades 1 to 3. Besides different therapy regimens and T cell quantification methods, a relatively small cohort size and heterogeneous composition due to the impeded procurement of paired STS samples is a limitation that may lead to these overall inconclusive findings. Especially the tumor location was shown to have profound impact on treatment strategies and clinical outcome. Compared to STS of the internal trunk, trunk wall, and head and neck, STS of the limbs was shown to display lower incidence of local relapse (47).

By comparing the T cell landscape of STS patients post-treatment versus pre-treatment, we found striking evidence that hyperthermic (chemo)radiotherapy not only modulates the pre-existing T cell landscape but, more importantly, re-programs it into a prognostically significant landscape. While the pre-existing T cell infiltration had no impact on the DFS of STS patients, high peritumoral and intratumoral CD3^+^ and high peritumoral CD8^+^ T cell levels after multimodal therapy were significantly associated with prolonged DFS. These results are in line with the observations of Issels et al. in a cohort of STS patients treated with neoadjuvant CTX with or without RHT (20). This is particularly relevant since most studies investigating the STS immune TME focus on pre-treatment immune landscapes or combine patients that received different therapy regimens. Since the prognostic value of T cell infiltrates in STS is rather inconclusive across different studies (48), our findings suggest that future study designs should differentiate between treatment regimens and consider the added value of the post-treatment immune TME. Furthermore, patients exhibiting a high post- to pre-treatment T cell ratio had a strong trend towards prolonged DFS, emphasizing the immunomodulatory properties of conventional anticancer treatments and its clinical relevance.

Besides the frequency of tumor-infiltrating T cells, their polarization and functional orientation is crucial in determining efficacy of the antitumor immune response, clinical outcome, and treatment response. Especially Th cells can adapt a wide spectrum of opposing functional properties. To explore the Th polarization in-situ, multiplexing is necessary to properly define the four main subsets of T-bet^+^ CD3^+^ Th1, GATA3^+^ CD3^+^ Th2, RORγT^+^ CD3^+^ Th17, and FoxP3^+^ CD3^+^ Tregs. Hence, many studies focus on peripheral blood or transcriptomic data, and few studies successfully analyzing Th cell composition *in-situ* are investigating tumor entities with higher incidence, such as lung and colorectal cancer (49–51). To the best of our knowledge, no in-depth characterization of the STS-infiltrating Th cell composition was reported so far. In this study, we observed a heavily Th2-dominated pre-treatment Th cell landscape, accompanied by moderate Th1 and Treg proportions and only a small fraction of Th17 polarized cells. Upon therapy, reduction of Th2 cell density was observed, resulting in a similar abundance as Th1 polarized T cells. Generally, Th2 cells are associated with protumor function and thus poor survival, although we did not observe relevant association with clinical outcome. Interestingly, Abeshouse et al. found that an elevated Th2 signature was linked to worse survival only in dedifferentiated liposarcoma, but not in other STS subtypes (52). Furthermore, STS are usually characterized by a high abundance of M2 macrophages, which can be induced by the Th2-associated cytokines IL-4 and IL-13, further promoting the immunosuppressive immune contexture of STS (53, 54). Therapy-induced reduction of Th2 infiltration suggests that multimodal therapy may induce beneficial changes in the Th cell landscape leading to a less suppressive immune TME.

While CD8^+^ T cells are usually characterized by their potent tumor killing capacity, several factors such as prolonged antigen exposure and an immunosuppressive environment can facilitate T cell dysfunction. These T cells are commonly defined by expression of immune checkpoint molecules such as PD-1 and LAG-3, although activated T cells can also exhibit checkpoint expression (55, 56). Thus, it is not surprising that heavily conflicting results regarding the association with clinical outcome were described. While Kim et al. found that both PD-1 and PD-L1 expression were associated with advanced clinicopathological parameters and worse OS of STS patients, other studies reported no clinical significance (57, 58). In a cohort of dedifferentiated liposarcoma, PD-1 expression assessed by transcriptomic profiling was linked to longer recurrence-free survival (59). When differentiating between mutation- or copy number-associated STS subtypes and translocation-driven subtypes, Dancsok et al. found that PD-1 expression was associated with worse survival in the former group, while it had no effect in the latter (40). These conflicting results may once again partly arise from heterogeneous cohorts and different treatment regimens. This is supported by our findings, demonstrating that the positive prognostic impact of PD-1^+^ CD8^+^ T cells was limited to the post-treatment sample, and significant only in the intratumoral area.

By employing an unsupervised hierarchical clustering approach, we identified three distinct groups of T cell-low, -intermediate, and -high patients. While the association with DFS is challenging due to low patient numbers in these subgroups, a trend towards shorter DFS in patients of the T cell-low cluster was found. In accordance with the clinical association of CD3^+^ and CD8^+^ T cell densities, this was only observed in post-treatment samples. Similar results were found in other studies, although the clustering is mostly based on transcriptomic data. For example, the five sarcoma immune classes identified by Petitprez et al. in the TCGA-SARC cohort exhibit distinct immune cell features and are strongly associated with OS (10). As these results are based on transcriptomic data of a total of 213 STS patients, signatures of not only T cells but also myeloid and stromal cells are included, allowing a more distinct classification. Few studies also employed hierarchical clustering for IHC data. In a cohort of undifferentiated pleomorphic sarcoma, classical IHC staining of T cells, B cells, and macrophages revealed three distinct immune clusters which showed significant differences in regard to OS (60). Thus, our obtained T cell-based clustering may be extended by staining for additional immune cell markers and can serve to support traditional transcriptomic-based clustering.

In addition to the frequency and functional orientation, the distribution of T cells plays a crucial role in antitumor immunity. For example, the IHC-based Immunoscore represents a tumor classification system of reliable prognostic significance that was developed and validated in colorectal cancer (18, 61). It is based on the frequency of CD3^+^ and CD8^+^ T cells in both tumor center and invasive margin and thus also recognizes excluded tumor immune landscapes resulting in an intermediate Immunoscore. Excluded tumors are characterized by T cell accumulations in the invasive margin and a largely void tumor center. Very little is known about the immune infiltration of the STS margin, which was assessed as the peritumoral tissue in this study. Utilizing IHC, Sorbye et al. observed no significant correlation between peritumoral CD3^+^ and CD8^+^ T cells and disease-specific survival in a mixed cohort of therapy-naïve and neoadjuvantly treated patients (62). However, we demonstrated that an increased post-therapy frequency of peritumoral CD3^+^ and CD8^+^ T cells was significantly associated with prolonged DFS in our STS cohort. Most strikingly, a higher intratumoral:peritumoral CD8^+^ T cell ratio was linked to significantly improved DFS and a decreased hazard ratio in a multivariate Cox PH model. To the best of our knowledge, our study provides the first evidence for the prognostic relevance of CD8^+^ T cell spatial distribution in STS. Of note, this finding is based on the post-treatment CD8^+^ T cell landscape and may not be reproducible in a therapy-naïve immune TME as pre-treatment T cell infiltration failed to provide prognostic value.

In summary, we found that post-treatment samples generally displayed lower T cell densities compared to pre-treatment biopsies, although these differences were not statistically significant. While a higher CD8^+^ and CD3^+^ T cell infiltration in the post-treatment specimen was significantly associated with improved DFS, these frequencies did not show clinical significance in the biopsy obtained prior to therapy. Moreover, spatial analysis revealed that an increased ratio of intra- to peritumoral CD8^+^ T cells was linked to significantly longer DFS and emerged as an independent prognostic factor for a lower risk of recurrence in a multivariate setting. In conclusion, these results emphasize the relevance of the post-therapy T cell landscape and the outstanding predictive value of its spatial organization. While a validation using an independent patient cohort would strengthen our findings, no publicly accessible data set is currently available that fits our unique study design, including the comparison of matched pre- and post-therapy samples as well as peritumoral and intratumoral regions. Altogether our analysis revealed that the functional orientation of STS-infiltrating T cells acquired a minor role, as the survival benefit was mainly linked to the abundance and spatial distribution of CD3^+^ and CD8^+^ T cells, while the expression of activation markers like GrzB or 4-1BB by CD8^+^ T cells or the polarization of Th cells did not significantly affect clinical outcome. Thus, considering the feasibility of the challenging multiplex technique, routine staining for CD3 and CD8 may suffice to effectively stratify patients and select the most beneficial treatment modalities. As the Immunoscore has proven extremely accurate in predicting survival in colorectal cancer (61), a similar classification system may yield promising results for STS patients. An enhanced understanding of the immunomodulatory effects of standard therapies is crucial for developing novel strategies to improve the efficacy of anticancer treatments and increase the long-term outcome of STS patients.

## Data availability statement

The original contributions presented in the study are included in the article/**Supplementary Material**. Further inquiries can be directed to the corresponding author.

## Ethics statement

The studies involving human participants were reviewed and approved by Ethics Committee of the Medical Faculty of the University of Tuebingen. The patients/participants provided their written informed consent to participate in this study.

## Author contributions

LR, FE, and MS contributed to conception. FE and HB selected and provided patient material. VW, HB, and KJ provided pathological assistance. LR and AR performed all experiments, analyzed, and visualized data. LR, AR, VP, and FE wrote and reviewed the original draft of the manuscript. VW, RW, HB, KJ, and MS reviewed and edited the manuscript. All authors contributed to the article and approved the submitted version.
