# Supplementary material for: Prognostic impact of the post-treatment T cell composition and spatial organization in soft tissue sarcoma patients treated with neoadjuvant hyperthermic radio(chemo)therapy
Source: Front Immunol. 2023 May 16;14:1185197. doi: 10.3389/fimmu.2023.1185197 (PMC10228739; doi:10.3389/fimmu.2023.1185197)
Supplement: Supplementary file 1 [file DataSheet_1.docx]

Supplementary Material

**Prognostic impact of the post-treatment T cell composition and spatial organization in soft tissue sarcoma patients treated with neoadjuvant hyperthermic radio(chemo)therapy**


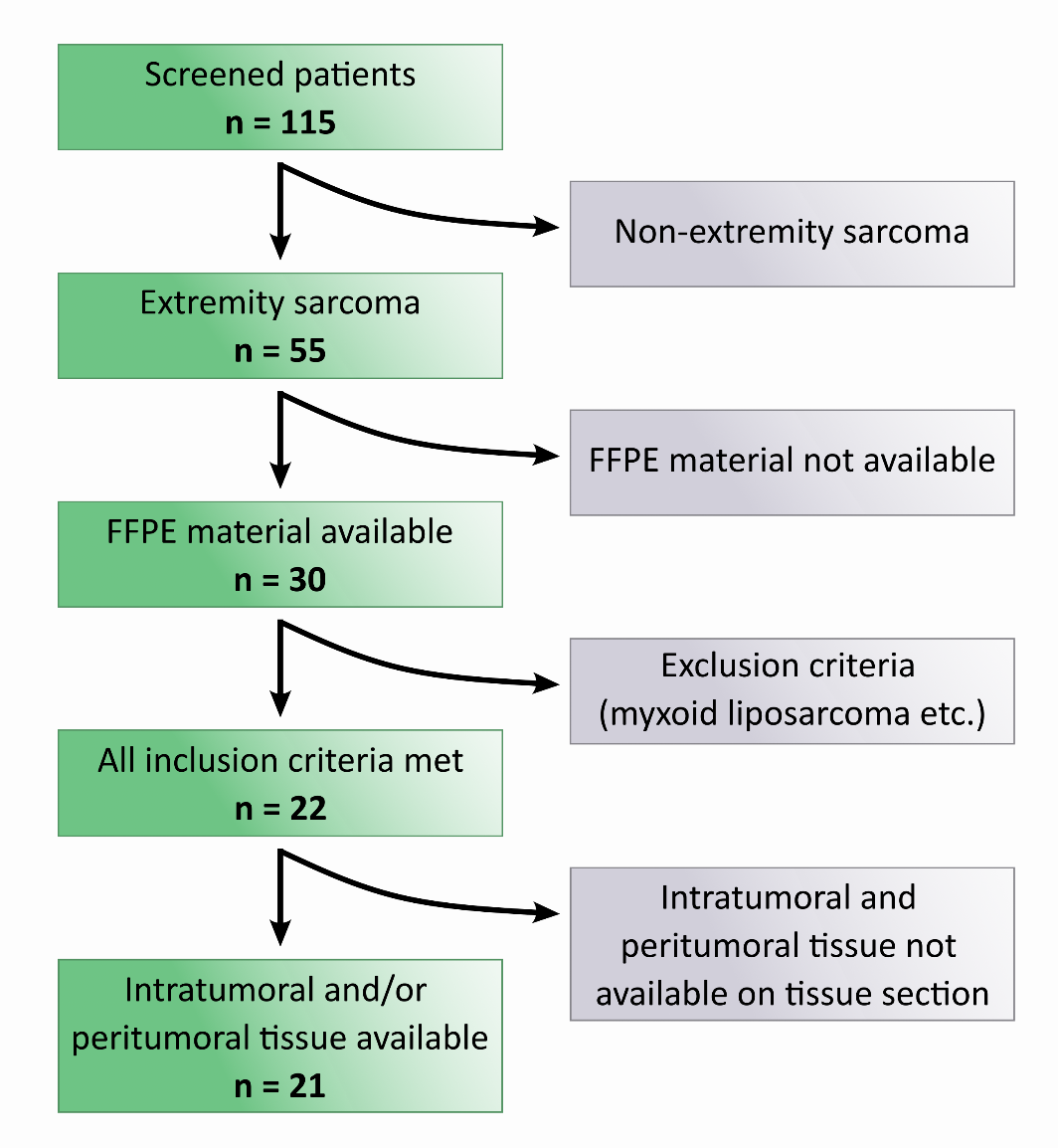


**Supplementary Figure 1.** **Selection of patients for the final soft-tissue sarcoma (STS) cohort.** Only extremity STS with available Formalin-fixed, Paraffin-embedded (FFPE) tumor material were selected for the cohort. Furthermore, STS subtypes with different clinical behavior (e.g. myxoid liposarcoma) were excluded. Only patients of whom at least one of the regions of interest - intratumoral and peritumoral tissue - was available were analyzed.

**Supplementary Table 1.** **Antibodies used for the 7-color mIHC panels.**

| **Antibody** | **Dilution** | **Incubation time [min]** | **Clone** | **Supplier** | **Order #** |
| --- | --- | --- | --- | --- | --- |
| Anti-4-1BB | 1:50 | 32 | D2Z4Y | Cell Signaling Technology, Inc. | 34594 |
| Anti-CD3 | prediluted | 32 | 2GV6 | Ventana Medical Systems, Inc. | 790-4341 |
| Anti-CD8 | prediluted | 32 | SP57 | Ventana Medical Systems, Inc. | 790-4460 |
| Anti-FoxP3 | 1:50 | 32 | 236A/E7 | Abcam plc. | ab20034 |
| Anti-Ki67 | 1:100 | 32 | MIB-1 | Agilent Technologies, Inc. | M7240 |
| Anti-GATA3 | 1:75 | 32 | D13C9 | Cell Signaling Technology, Inc. | 5852 |
| Anti-GrzB | 1:100 | 32 | Grb-7 | Agilent Technologies, Inc. | M7235 |
| Anti-LAG-3 | 1:50 | 32 | D2G40 | Cell Signaling Technology, Inc. | 15372 |
| Anti-PD-1 | prediluted | 60 | NAT105 | Ventana Medical Systems, Inc. | 760-4895 |
| Anti-RORγT | 1:100 | 60 | 6F3.1 | Merck KGaA | MABF81 |
| Anti-T-bet | 1:100 | 32 | D6N8B | Cell Signaling Technology, Inc. | 13232 |

**
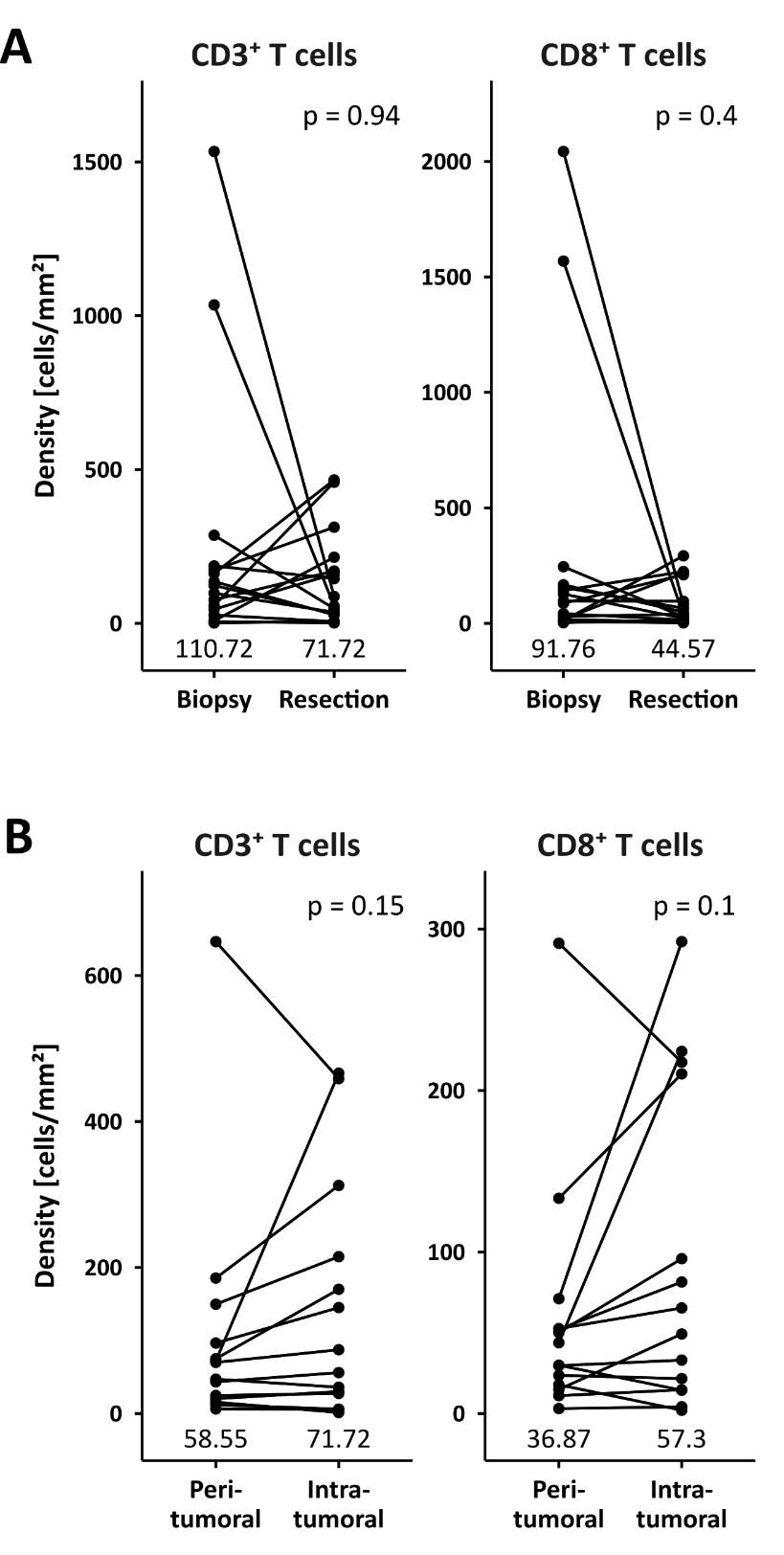
**

**Supplementary Figure 2.** **Analysis of CD3^+^ and CD8^+^ T cell infiltration in paired STS samples.** (A-B) Densities [cells/mm²] of CD3^+^ and CD8^+^ T cells compared between paired intratumoral biopsy-resection (A) and paired intratumoral-peritumoral resection samples (B). Paired Wilcoxon test was performed and p-values ≤ 0.05 were considered significant.

**Supplementary Table 2.** **Comparison of T cell infiltrates between grade 2 and grade 3 STS.** Median value and interquartile range (IQR) of densities and proportions of all T cell subtypes compared between grade 2 and grade 3 STS. Unpaired Wilcoxon test was performed and p-values ≤ 0.05 were considered significant.

| **Phenotype** | **Grade 2** | | **Grade 3** | | **Wilcoxon p-value** |
| --- | --- | --- | --- | --- | --- |
|  | **Median** | **IQR** | **Median** | **IQR** |  |
| **Biopsy sample - intratumoral tissue** | | | | | |
| Density CD3 all | 67.168 | 12.941-132.721 | 164.741 | 91.41-183.265 | 0.1051 |
| Density CD3 FoxP3 | 15.866 | 1.494-23.034 | 31.475 | 12.59-47.604 | 0.0630 |
| Density CD3 GATA3 | 21.549 | 5.732-65.038 | 95.676 | 33.114-117.861 | 0.1051 |
| Density CD3 RORγT | 1.703 | 0.636-2.905 | 4.005 | 3.058-7.817 | 0.0115 |
| Density CD3 T-bet | 8.883 | 3.453-31.208 | 29.827 | 20.844-44.951 | 0.0524 |
| Density CD8 all | 57.614 | 13.193-156.921 | 93.405 | 53.179-152.38 | 0.3150 |
| Density CD8 4-1BB | 1.756 | 0.152-4.746 | 2.021 | 0.77-3.253 | 0.4359 |
| Density CD8 GrzB | 0.9 | 0.416-2.747 | 5.804 | 3.696-10.064 | 0.0089 |
| Density CD8 Ki67 | 7.271 | 2.027-48.83 | 23.669 | 15.452-36.785 | 0.1903 |
| Density CD8 LAG-3 | 2.113 | 1.127-42.147 | 27.66 | 6.912-70.859 | 0.0524 |
| Density CD8 PD-1 | 15.952 | 1.324-47.541 | 19.977 | 14.754-50.872 | 0.3150 |
| Proportion CD8 4-1BB | 1.831 | 0.937-4.004 | 1.417 | 1.078-2.762 | 0.8534 |
| Proportion CD8 GrzB | 2.34 | 1.545-4.663 | 6.02 | 4.039-7.197 | 0.0147 |
| Proportion CD8 Ki67 | 21.637 | 11.996-31.071 | 24.457 | 22.544-27.638 | 0.6305 |
| Proportion CD8 LAG-3 | 8.608 | 3.081-10.905 | 21.47 | 10.849-32.558 | 0.0524 |
| Proportion CD8 PD-1 | 20.229 | 14.339-28.741 | 22.253 | 19.546-29.075 | 0.5787 |
| Proportion CD3 FoxP3 | 24.527 | 15.28-31.282 | 23.253 | 12.413-32.199 | 0.7959 |
| Proportion CD3 GATA3 | 78.76 | 50.89-80.234 | 73.788 | 56.642-79.01 | 0.6842 |
| Proportion CD3 RORγT | 4.246 | 2.044-11.907 | 3.437 | 1.928-7.136 | 0.3930 |
| Proportion CD3 T-bet | 41.686 | 28.282-59.646 | 32.796 | 28.616-46.947 | 0.7959 |
| **Resection sample - peritumoral tissue** | | | | | |
| Density CD3 all | 24.686 | 15.493-149.739 | 58.551 | 34.736-79.535 | 0.842 |
| Density CD3 FoxP3 | 1.807 | 0.334-6.816 | 4 | 1.419-5.643 | 0.549 |
| Density CD3 GATA3 | 7.745 | 4.588-41.907 | 20.276 | 7.263-32.422 | 0.842 |
| Density CD3 RORγT | 2.213 | 0.688-3.408 | 2.898 | 2.174-4.07 | 0.604 |
| Density CD3 T-bet | 8.347 | 5.1-58.343 | 13.387 | 7.365-19.536 | 0.905 |
| Density CD8 all | 23.598 | 14.886-71.09 | 36.867 | 25.689-51.89 | 0.780 |
| Density CD8 4-1BB | 0.686 | 0.19-4.425 | 0.849 | 0.605-1.665 | 0.905 |
| Density CD8 GrzB | 1.228 | 0.846-3.789 | 1.826 | 1.237-2.295 | 0.968 |
| Density CD8 Ki67 | 4.773 | 3.046-10.914 | 3.277 | 2.815-5.568 | 0.447 |
| Density CD8 LAG-3 | 3.975 | 2.717-5.342 | 6.943 | 3.111-10.413 | 0.661 |
| Density CD8 PD-1 | 6.382 | 4.535-17.109 | 9.786 | 5.115-14.048 | 0.842 |

**Supplementary Table 2.** Continued from page 4.

| Proportion CD8 4-1BB | 2.908 | 1.724-4.301 | 3.149 | 2.26-3.626 | 0.842 |
| --- | --- | --- | --- | --- | --- |
| Proportion CD8 GrzB | 8.319 | 5.682-10.345 | 5.28 | 3.608-8.792 | 0.315 |
| Proportion CD8 Ki67 | 17.348 | 13.889-27.586 | 9.565 | 6.18-13.668 | 0.028 |
| Proportion CD8 LAG-3 | 15.278 | 10.641-30.108 | 19.403 | 11.851-26.074 | 0.905 |
| Proportion CD8 PD-1 | 30.454 | 19.216-34.384 | 22.477 | 16.969-35.968 | 0.968 |
| Proportion CD3 FoxP3 | 9.171 | 7.616-14.789 | 12.066 | 9.136-14.78 | 0.604 |
| Proportion CD3 GATA3 | 55.298 | 49.752-85.572 | 73.715 | 57.645-83.531 | 0.497 |
| Proportion CD3 RORγT | 4.512 | 3.311-17.33 | 11.952 | 8.835-15.416 | 0.315 |
| Proportion CD3 T-bet | 57.914 | 50.311-68.31 | 43.789 | 26.562-59.187 | 0.211 |
| **Resection sample - intratumoral tissue** | | | | | |
| Density CD3 all | 30.138 | 6.018-170.184 | 145.188 | 71.719-236.494 | 0.142 |
| Density CD3 FoxP3 | 3.235 | 0.649-7.719 | 29.589 | 14.226-52.53 | 0.091 |
| Density CD3 GATA3 | 10.373 | 3.317-24.264 | 25.973 | 9.591-81.717 | 0.210 |
| Density CD3 RORγT | 1.281 | 0.212-3.116 | 4.143 | 1.74-8.025 | 0.299 |
| Density CD3 T-bet | 7.387 | 2.2-23.118 | 25.088 | 14.752-38.282 | 0.299 |
| Density CD8 all | 27.904 | 14.691-95.884 | 65.374 | 36.491-145.9 | 0.470 |
| Density CD8 4-1BB | 0.487 | 0.151-1.357 | 1.181 | 0.567-4.13 | 0.351 |
| Density CD8 GrzB | 1.798 | 1.196-3.806 | 1.67 | 1.03-9.715 | 0.758 |
| Density CD8 Ki67 | 4.963 | 1.363-36.527 | 9.01 | 3.759-19.852 | 0.758 |
| Density CD8 LAG-3 | 9.378 | 0.971-15.523 | 9.591 | 6.09-51.358 | 0.470 |
| Density CD8 PD-1 | 6.393 | 1.212-11.565 | 12.461 | 7.106-50.636 | 0.252 |
| Proportion CD8 4-1BB | 1.724 | 1.281-3.753 | 2.074 | 1.478-2.727 | 0.918 |
| Proportion CD8 GrzB | 6.444 | 3.448-9.278 | 5.172 | 3.365-6.843 | 0.837 |
| Proportion CD8 Ki67 | 18.487 | 9.278-32.886 | 12.904 | 8.978-16.111 | 0.351 |
| Proportion CD8 LAG-3 | 23.276 | 16.189-27.768 | 29.003 | 10.789-35.137 | 0.681 |
| Proportion CD8 PD-1 | 23.529 | 12.061-33.45 | 27.88 | 19.538-33.127 | 0.470 |
| Proportion CD3 FoxP3 | 17.021 | 13.953-18.519 | 26.856 | 15.438-53.786 | 0.299 |
| Proportion CD3 GATA3 | 50.376 | 10.638-71.318 | 50.87 | 30.663-61.163 | 0.681 |
| Proportion CD3 RORγT | 8.333 | 3.918-15.521 | 4.62 | 2.232-13.322 | 0.606 |
| Proportion CD3 T-bet | 63.48 | 47.287-70.722 | 38.845 | 20.414-48.664 | 0.142 |

**Supplementary Table 3.** **Comparison of T cell infiltrates between STS of smaller and larger size.** Median value and interquartile range (IQR) of densities and proportions of all T cell subtypes compared between STS smaller than 10 cm maximum diameter and larger equal 10 cm maximum diameter. Unpaired Wilcoxon test was performed and p-values ≤ 0.05 were considered significant.

| **Phenotype** | **Size < 10 cm** | | **Size ≥ 10 cm** | | **Wilcoxon p-value** |
| --- | --- | --- | --- | --- | --- |
|  | **Median** | **IQR** | **Median** | **IQR** |  |
| **Biopsy sample - intratumoral tissue** | | | | | |
| Density CD3 all | 161.85 | 26.686-173.089 | 98.185 | 62.698-161.266 | 1.000 |
| Density CD3 FoxP3 | 23.564 | 2.711-43.093 | 14.536 | 9.251-25.661 | 0.503 |
| Density CD3 GATA3 | 98.534 | 15.369-119.035 | 28.607 | 22.523-85 | 0.824 |
| Density CD3 RORγT | 4.301 | 1.809-8.917 | 2.464 | 1.393-3.339 | 0.175 |
| Density CD3 T-bet | 28.999 | 7.427-41.149 | 20.483 | 13.592-41.238 | 0.941 |
| Density CD8 all | 86.62 | 17.49-142.656 | 96.898 | 40.752-161.267 | 0.412 |
| Density CD8 4-1BB | 2.015 | 0.386-3.379 | 1.599 | 0.468-4.016 | 0.882 |
| Density CD8 GrzB | 3.392 | 0.525-10.501 | 2.919 | 2.138-5.959 | 0.941 |
| Density CD8 Ki67 | 19.515 | 3.644-37.199 | 23.248 | 5.986-53.255 | 0.603 |
| Density CD8 LAG-3 | 9.585 | 1.911-32.143 | 6.846 | 3.203-57.347 | 0.656 |
| Density CD8 PD-1 | 17.474 | 2.54-32.491 | 28.909 | 11.49-70.396 | 0.295 |
| Proportion CD8 4-1BB | 2.108 | 1.412-3.901 | 1.303 | 0.766-1.831 | 0.295 |
| Proportion CD8 GrzB | 5.487 | 2.861-5.916 | 2.961 | 1.263-5.984 | 0.412 |
| Proportion CD8 Ki67 | 22.586 | 19.88-26.076 | 27.905 | 19.474-32.559 | 0.456 |
| Proportion CD8 LAG-3 | 10.926 | 10.638-22.532 | 9.855 | 6.093-31.266 | 0.882 |
| Proportion CD8 PD-1 | 19.394 | 13.855-22.776 | 29.835 | 20.191-35.051 | 0.046 |
| Proportion CD3 FoxP3 | 20.366 | 18.018-32.998 | 28.487 | 9.508-30.817 | 0.456 |
| Proportion CD3 GATA3 | 78.087 | 66.393-79.317 | 70.746 | 48.917-83.497 | 1.000 |
| Proportion CD3 RORγT | 7.206 | 3.464-11.765 | 3.41 | 1.588-5.66 | 0.295 |
| Proportion CD3 T-bet | 30.62 | 28.736-44.562 | 42.249 | 28.241-57.175 | 0.710 |
| **Resection sample - peritumoral tissue** | | | | | |
| Density CD3 all | 57.752 | 13.575-158.697 | 47.075 | 22.497-78.524 | 1.00 |
| Density CD3 FoxP3 | 5.058 | 0.426-13.127 | 1.846 | 1.012-5.039 | 0.78 |
| Density CD3 GATA3 | 24.003 | 4.57-35.214 | 10.359 | 5.828-34.167 | 0.84 |
| Density CD3 RORγT | 2.563 | 2.091-3.641 | 2.947 | 0.919-4.092 | 0.72 |
| Density CD3 T-bet | 15.459 | 5.106-31.377 | 8.347 | 6.116-18.279 | 0.49 |
| Density CD8 all | 36.746 | 14.606-86.64 | 29.889 | 19.932-51.627 | 0.97 |
| Density CD8 4-1BB | 2.056 | 0.167-3.899 | 0.686 | 0.43-1.306 | 0.97 |
| Density CD8 GrzB | 2.176 | 0.58-3.324 | 1.547 | 1.138-2.293 | 0.90 |
| Density CD8 Ki67 | 3.949 | 1.951-7.05 | 4.446 | 3.113-8.707 | 0.54 |
| Density CD8 LAG-3 | 4.853 | 2.268-16.909 | 4.404 | 2.757-9.142 | 0.78 |
| Density CD8 PD-1 | 10.218 | 3.275-20.671 | 7.632 | 4.687-12.604 | 1.00 |

**Supplementary Table 3.** Continued from page 6.

| Proportion CD8 4-1BB | 3.099 | 2.492-4.782 | 3.073 | 1.492-3.441 | 0.60 |
| --- | --- | --- | --- | --- | --- |
| Proportion CD8 GrzB | 6.929 | 5.449-10.591 | 5.682 | 3.67-9.469 | 0.72 |
| Proportion CD8 Ki67 | 13.022 | 8.3-14.255 | 17.348 | 9.083-23.729 | 0.35 |
| Proportion CD8 LAG-3 | 20.653 | 14.007-27.479 | 18.361 | 9.803-26.889 | 0.78 |
| Proportion CD8 PD-1 | 27.26 | 22.645-32.641 | 21.429 | 15.738-35.829 | 0.90 |
| Proportion CD3 FoxP3 | 11.257 | 7.338-19.065 | 11.34 | 9.108-14.161 | 0.97 |
| Proportion CD3 GATA3 | 62.577 | 51.302-82.321 | 66.943 | 53.026-85.148 | 0.97 |
| Proportion CD3 RORγT | 9.373 | 4.374-16.477 | 11.34 | 4.473-16.67 | 0.78 |
| Proportion CD3 T-bet | 54.112 | 45.909-61.705 | 51.987 | 29.624-65.562 | 0.90 |
| **Resection sample - intratumoral tissue** | | | | | |
| Density CD3 all | 135.446 | 37.295-349.054 | 61.67 | 29.469-149.02 | 0.44180 |
| Density CD3 FoxP3 | 18.654 | 3.6-49.299 | 3.23 | 1.947-17.862 | 0.38228 |
| Density CD3 GATA3 | 25.118 | 10.026-57.556 | 9.591 | 6.853-14.662 | 0.27863 |
| Density CD3 RORγT | 1.621 | 1.198-4.861 | 3.032 | 1.193-7.897 | 0.79845 |
| Density CD3 T-bet | 33.86 | 3.334-91.202 | 11.481 | 7.375-19.993 | 0.38228 |
| Density CD8 all | 121.703 | 21.971-219.21 | 44.573 | 19.885-69.396 | 0.57374 |
| Density CD8 4-1BB | 3.964 | 0.596-8.457 | 0.425 | 0.277-0.768 | 0.19487 |
| Density CD8 GrzB | 2.802 | 1.347-15.573 | 1.339 | 1.084-2.784 | 0.44180 |
| Density CD8 Ki67 | 17.074 | 2.926-67.984 | 5.479 | 2.072-9.449 | 0.57374 |
| Density CD8 LAG-3 | 43.839 | 6.054-83.062 | 8.537 | 3.735-11.046 | 0.27863 |
| Density CD8 PD-1 | 42.439 | 4.731-85.398 | 7.263 | 3.878-11.789 | 0.44180 |
| Proportion CD8 4-1BB | 3.557 | 2.926-4.337 | 1.215 | 1-1.54 | 0.00062 |
| Proportion CD8 GrzB | 6.521 | 4.649-9.439 | 4.787 | 1.663-7.636 | 0.57374 |
| Proportion CD8 Ki67 | 17.12 | 13.375-32.04 | 10.169 | 6.618-19.286 | 0.19487 |
| Proportion CD8 LAG-3 | 28.386 | 25.861-37.356 | 13.711 | 10.12-22.92 | 0.13038 |
| Proportion CD8 PD-1 | 34.089 | 22.244-35.335 | 16.564 | 11.167-28.304 | 0.02813 |
| Proportion CD3 FoxP3 | 18.513 | 14.004-31.984 | 18.507 | 10.251-48.044 | 1.00000 |
| Proportion CD3 GATA3 | 61.163 | 21.75-72.045 | 46.499 | 16.846-52.997 | 0.38228 |
| Proportion CD3 RORγT | 4.269 | 2.565-10.27 | 9.662 | 5.282-16.698 | 0.44180 |
| Proportion CD3 T-bet | 51.957 | 35.141-73.254 | 47.684 | 24.422-64.426 | 0.72090 |
